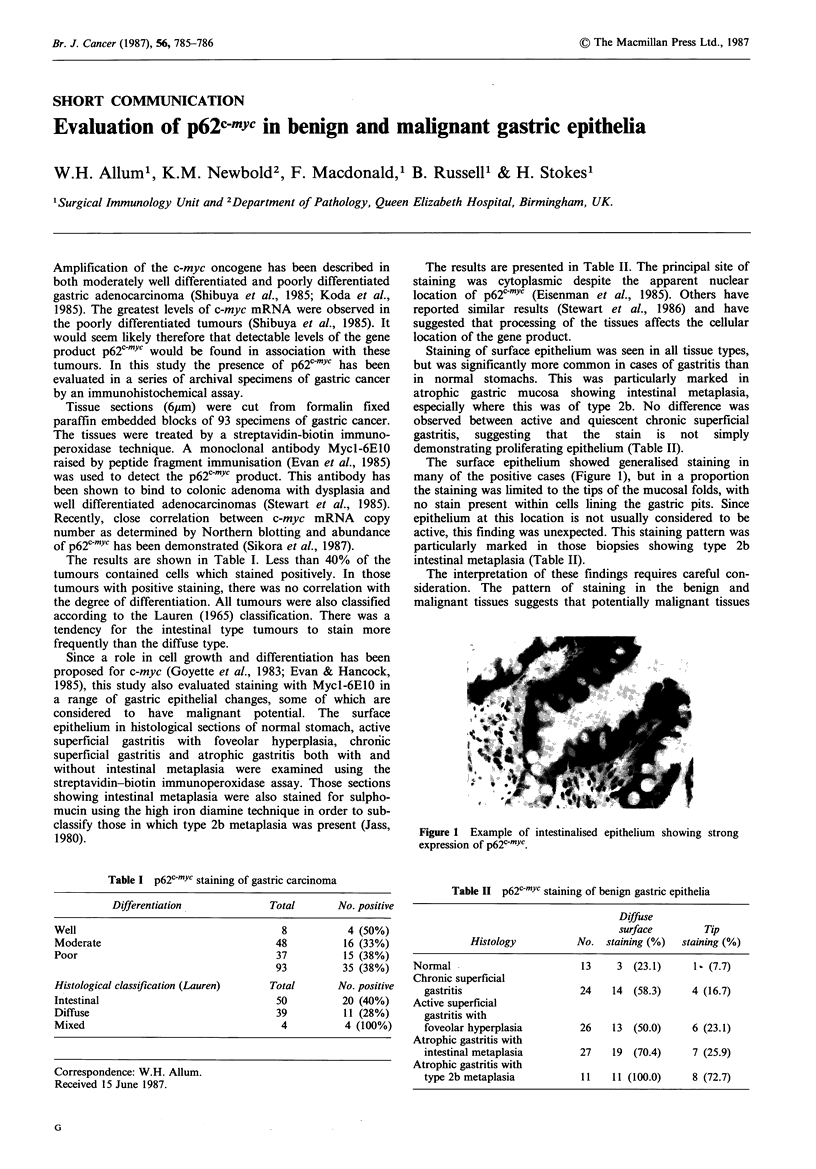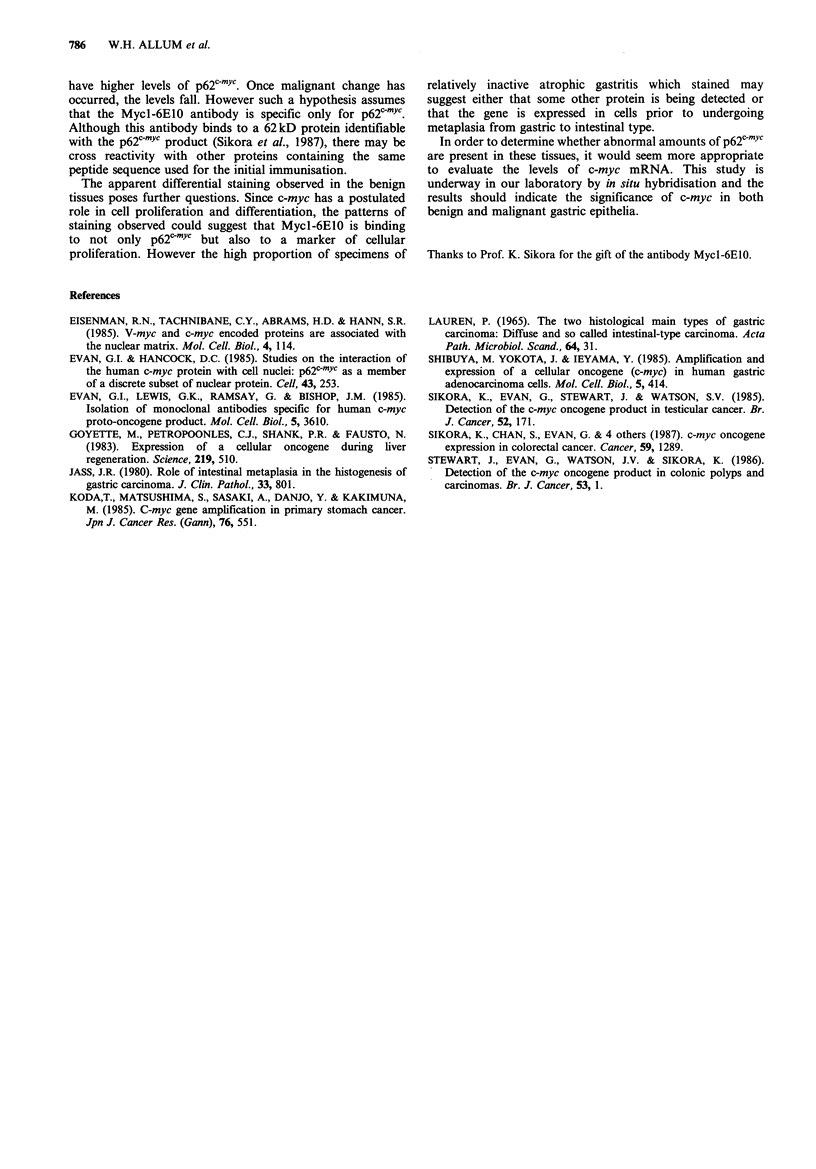# Evaluation of p62c-myc in benign and malignant gastric epithelia.

**DOI:** 10.1038/bjc.1987.288

**Published:** 1987-12

**Authors:** W. H. Allum, K. M. Newbold, F. Macdonald, B. Russell, H. Stokes

**Affiliations:** Surgical Immunology Unit, Queen Elizabeth Hospital, Birmingham, UK.

## Abstract

**Images:**


Br. J. Cancer (1987), 56, 785–786 © The Macmillan Press Ltd., 1987

SHORT COMMUNICATION

### Evaluation of p62$^{c\text{-}myc}$ in benign and malignant gastric epithelia

W.H. Allum[1], K.M. Newbold[2], F. Macdonald,[1] B. Russell[1] & H. Stokes[1]

[1]*Surgical Immunology Unit and* [2]*Department of Pathology, Queen Elizabeth Hospital, Birmingham, UK.*

Amplification of the c-*myc* oncogene has been described in both moderately well differentiated and poorly differentiated gastric adenocarcinoma (Shibuya *et al.*, 1985; Koda *et al.*, 1985). The greatest levels of c-*myc* mRNA were observed in the poorly differentiated tumours (Shibuya *et al.*, 1985). It would seem likely therefore that detectable levels of the gene product p62$^{c\text{-}myc}$ would be found in association with these tumours. In this study the presence of p62$^{c\text{-}myc}$ has been evaluated in a series of archival specimens of gastric cancer by an immunohistochemical assay.

Tissue sections (6μm) were cut from formalin fixed paraffin embedded blocks of 93 specimens of gastric cancer. The tissues were treated by a streptavidin-biotin immunoperoxidase technique. A monoclonal antibody Mycl-6E10 raised by peptide fragment immunisation (Evan *et al.*, 1985) was used to detect the p62$^{c\text{-}myc}$ product. This antibody has been shown to bind to colonic adenoma with dysplasia and well differentiated adenocarcinomas (Stewart *et al.*, 1985). Recently, close correlation between c-*myc* mRNA copy number as determined by Northern blotting and abundance of p62$^{c\text{-}myc}$ has been demonstrated (Sikora *et al.*, 1987).

The results are shown in Table I. Less than 40% of the tumours contained cells which stained positively. In those tumours with positive staining, there was no correlation with the degree of differentiation. All tumours were also classified according to the Lauren (1965) classification. There was a tendency for the intestinal type tumours to stain more frequently than the diffuse type.

Since a role in cell growth and differentiation has been proposed for c-*myc* (Goyette *et al.*, 1983; Evan & Hancock, 1985), this study also evaluated staining with Mycl-6E10 in a range of gastric epithelial changes, some of which are considered to have malignant potential. The surface epithelium in histological sections of normal stomach, active superficial gastritis with foveolar hyperplasia, chronic superficial gastritis and atrophic gastritis both with and without intestinal metaplasia were examined using the streptavidin–biotin immunoperoxidase assay. Those sections showing intestinal metaplasia were also stained for sulphomucin using the high iron diamine technique in order to subclassify those in which type 2b metaplasia was present (Jass, 1980).

**Table I** p62$^{c\text{-}myc}$ staining of gastric carcinoma

| *Differentiation* | *Total* | *No. positive* |
|---|---|---|
| Well | 8 | 4 (50%) |
| Moderate | 48 | 16 (33%) |
| Poor | 37 | 15 (38%) |
| | 93 | 35 (38%) |
| *Histological classification (Lauren)* | *Total* | *No. positive* |
| Intestinal | 50 | 20 (40%) |
| Diffuse | 39 | 11 (28%) |
| Mixed | 4 | 4 (100%) |

Correspondence: W.H. Allum.
Received 15 June 1987.

The results are presented in Table II. The principal site of staining was cytoplasmic despite the apparent nuclear location of p62$^{c\text{-}myc}$ (Eisenman *et al.*, 1985). Others have reported similar results (Stewart *et al.*, 1986) and have suggested that processing of the tissues affects the cellular location of the gene product.

Staining of surface epithelium was seen in all tissue types, but was significantly more common in cases of gastritis than in normal stomachs. This was particularly marked in atrophic gastric mucosa showing intestinal metaplasia, especially where this was of type 2b. No difference was observed between active and quiescent chronic superficial gastritis, suggesting that the stain is not simply demonstrating proliferating epithelium (Table II).

The surface epithelium showed generalised staining in many of the positive cases (Figure 1), but in a proportion the staining was limited to the tips of the mucosal folds, with no stain present within cells lining the gastric pits. Since epithelium at this location is not usually considered to be active, this finding was unexpected. This staining pattern was particularly marked in those biopsies showing type 2b intestinal metaplasia (Table II).

The interpretation of these findings requires careful consideration. The pattern of staining in the benign and malignant tissues suggests that potentially malignant tissues

**Figure 1** Example of intestinalised epithelium showing strong expression of p62$^{c\text{-}myc}$.

**Table II** p62$^{c\text{-}myc}$ staining of benign gastric epithelia

| *Histology* | *No.* | *Diffuse surface staining* (%) | *Tip staining* (%) |
|---|---|---|---|
| Normal | 13 | 3 (23.1) | 1 (7.7) |
| Chronic superficial gastritis | 24 | 14 (58.3) | 4 (16.7) |
| Active superficial gastritis with foveolar hyperplasia | 26 | 13 (50.0) | 6 (23.1) |
| Atrophic gastritis with intestinal metaplasia | 27 | 19 (70.4) | 7 (25.9) |
| Atrophic gastritis with type 2b metaplasia | 11 | 11 (100.0) | 8 (72.7) |

have higher levels of p62$^{c\text{-}myc}$. Once malignant change has occurred, the levels fall. However such a hypothesis assumes that the Mycl-6E10 antibody is specific only for p62$^{c\text{-}myc}$. Although this antibody binds to a 62 kD protein identifiable with the p62$^{c\text{-}myc}$ product (Sikora *et al.*, 1987), there may be cross reactivity with other proteins containing the same peptide sequence used for the initial immunisation.

The apparent differential staining observed in the benign tissues poses further questions. Since c-*myc* has a postulated role in cell proliferation and differentiation, the patterns of staining observed could suggest that Mycl-6E10 is binding to not only p62$^{c\text{-}myc}$ but also to a marker of cellular proliferation. However the high proportion of specimens of relatively inactive atrophic gastritis which stained may suggest either that some other protein is being detected or that the gene is expressed in cells prior to undergoing metaplasia from gastric to intestinal type.

In order to determine whether abnormal amounts of p62$^{c\text{-}myc}$ are present in these tissues, it would seem more appropriate to evaluate the levels of c-*myc* mRNA. This study is underway in our laboratory by *in situ* hybridisation and the results should indicate the significance of c-*myc* in both benign and malignant gastric epithelia.

Thanks to Prof. K. Sikora for the gift of the antibody Mycl-6E10.

### References

EISENMAN, R.N., TACHNIBANE, C.Y., ABRAMS, H.D. & HANN, S.R. (1985). V-*myc* and c-*myc* encoded proteins are associated with the nuclear matrix. *Mol. Cell. Biol.*, **4**, 114.

EVAN, G.I. & HANCOCK, D.C. (1985). Studies on the interaction of the human c-*myc* protein with cell nuclei: p62$^{c\text{-}myc}$ as a member of a discrete subset of nuclear protein. *Cell*, **43**, 253.

EVAN, G.I., LEWIS, G.K., RAMSAY, G. & BISHOP, J.M. (1985). Isolation of monoclonal antibodies specific for human c-*myc* proto-oncogene product. *Mol. Cell. Biol.*, **5**, 3610.

GOYETTE, M., PETROPOONLES, C.J., SHANK, P.R. & FAUSTO, N. (1983). Expression of a cellular oncogene during liver regeneration. *Science*, **219**, 510.

JASS, J.R. (1980). Role of intestinal metaplasia in the histogenesis of gastric carcinoma. *J. Clin. Pathol.*, **33**, 801.

KODA,T., MATSUSHIMA, S., SASAKI, A., DANJO, Y. & KAKIMUNA, M. (1985). C-*myc* gene amplification in primary stomach cancer. *Jpn J. Cancer Res.* (*Gann*), **76**, 551.

LAUREN, P. (1965). The two histological main types of gastric carcinoma: Diffuse and so called intestinal-type carcinoma. *Acta Path. Microbiol. Scand.*, **64**, 31.

SHIBUYA, M. YOKOTA, J. & IEYAMA, Y. (1985). Amplification and expression of a cellular oncogene (c-*myc*) in human gastric adenocarcinoma cells. *Mol. Cell. Biol.*, **5**, 414.

SIKORA, K., EVAN, G., STEWART, J. & WATSON, S.V. (1985). Detection of the c-*myc* oncogene product in testicular cancer. *Br. J. Cancer*, **52**, 171.

SIKORA, K., CHAN, S., EVAN, G. & 4 others (1987). c-*myc* oncogene expression in colorectal cancer. *Cancer*, **59**, 1289.

STEWART, J., EVAN, G., WATSON, J.V. & SIKORA, K. (1986). Detection of the c-*myc* oncogene product in colonic polyps and carcinomas. *Br. J. Cancer*, **53**, 1.